# Morphometric Aortic Remodeling and Mid-Term Outcomes After TEVAR for Acute Stanford Type B Aortic Dissection: A Single-Center Retrospective Cohort Study

**DOI:** 10.3390/jcm15124714

**Published:** 2026-06-17

**Authors:** Kemal Eşref Erdoğan, Muhammet Fethi Sağlam, Murat Yücel, Emrah Uğuz, Servet Turgut, Halil Tekdemir, Mete Hıdıroğlu, Erol Şener

**Affiliations:** 1 Department of Cardiovascular Surgery, Ankara Yildirim Beyazit University, School of Medicine and Ankara Bilkent City Hospital, 06010 Ankara, Türkiye; dr.m.fethisaglam@gmail.com (M.F.S.); emrahuguz@gmail.com (E.U.); metetaha@hotmail.com (M.H.); erolsene@gmail.com (E.Ş.); 2 Department of Cardiovascular Surgery, Ankara Bilkent City Hospital, 06800 Ankara, Türkiye; dr_yucelmurat@hotmail.com; 3 Department of Cardiovascular Surgery, Kars Harakani State Hospital, 36100 Kars, Türkiye; drservetturgut@gmail.com; 4 Department of Radiology, Ankara Etlik City Hospital, 06170 Ankara, Türkiye; haltek04726@gmail.com

**Keywords:** aortic dissection, thoracic endovascular aortic repair, aortic remodeling, false lumen, true lumen, endoleak

## Abstract

**Objectives**: This study aimed to evaluate the impact of thoracic endovascular aortic repair (TEVAR) on aortic remodeling using CT angiography-based morphometric measurements and to examine associated mid-term clinical outcomes in patients with acute Stanford type B aortic dissection. **Methods**: This retrospective, single-center observational cohort study included 33 consecutive patients who underwent TEVAR for acute Stanford type B aortic dissection between January 2020 and January 2025. Preoperative and postoperative true lumen (TL), false lumen (FL), and descending aorta (DA) diameters were compared using paired *t*-tests after Shapiro–Wilk normality testing. Endoleak, reintervention, FL thrombosis, and mortality were analyzed. Univariable analyses identified factors associated with endoleak and reintervention. Spearman’s correlation assessed factors associated with morphometric remodeling response. **Results**: All 33 patients had acute Stanford type B dissection (mean time to intervention: 2.73 ± 3.86 days). Among 33 patients (81.8% male; mean age 53.6 ± 12.1 years), mean follow-up was 4.08 ± 1.66 years. TEVAR induced a significant aortic remodeling response: TL diameter increased from 9.55 ± 5.91 mm to 28.30 ± 5.49 mm (+18.76 ± 8.83 mm; *p* < 0.001) and FL diameter decreased from 33.39 ± 6.76 mm to 11.48 ± 8.97 mm (−21.91 ± 9.53 mm; *p* < 0.001), while DA diameter remained stable (42.94 ± 6.90 vs. 42.03 ± 9.46 mm; *p* = 0.323). Complete FL thrombosis was achieved in 19 patients (57.6%). Endoleak occurred in nine patients (27.3%); Zone 2 landing was significantly associated with endoleak (54.5% vs. 13.6%, *p* = 0.033). Secondary intervention was required in 13 patients (39.4%). Overall mortality was 12.1%. Narrower preoperative TL was strongly associated with greater TL expansion (Spearman r = −0.724, *p* < 0.001); longer stent–graft coverage was associated with greater TL gain (r = +0.522, *p* = 0.002). **Conclusions**: TEVAR induced clinically meaningful aortic remodeling in acute Stanford type B dissection without progressive aortic enlargement. A narrower baseline TL and longer stent–graft coverage were associated with greater remodeling benefit. Zone 2 deployment was significantly associated with higher endoleak rates, underscoring the value of careful preprocedural planning and systematic long-term imaging surveillance.

## 1. Introduction

Aortic dissection remains one of the most critical pathologies in cardiovascular surgery, associated with high short-term mortality and rapid clinical deterioration [1]. Stanford type B aortic dissection, confined to the descending thoracic aorta, is managed with optimal medical therapy in uncomplicated cases; however, endovascular intervention is increasingly indicated in the presence of malperfusion syndrome, threatening rupture, refractory hypertension, or rapid aortic expansion [2,3].

Thoracic endovascular aortic repair (TEVAR) has become the preferred treatment for complicated Stanford type B aortic dissection, with perioperative morbidity and mortality considerably lower than open surgical repair [2,4]. The primary biomechanical goal of TEVAR is to seal the primary intimal tear, restore true lumen (TL) flow, and promote false lumen (FL) thrombosis and regression—a process collectively described as ‘aortic remodeling’ [4]. Favorable remodeling, defined as TL expansion and FL regression without progressive aortic enlargement, is recognized as a key surrogate marker of technical success and long-term aortic stability [5].

However, the remodeling response following TEVAR is neither predictable nor homogeneous. Distal re-entry foci, unfavorable stent–graft positioning, preoperative FL morphology, aortic wall calcification, and morphological complexity may all limit this process [6]. Trials such as INSTEAD-XL, ADSORB, and the VIRTUE Registry have established the remodeling benefit of TEVAR; however, standardized morphometric quantification using multiplanar CT angiography reconstruction remains limited in real-world series [7,8]. The present study draws on our center’s prior endovascular experience in acute thoracic aortic syndromes [9].

The present study, therefore, aimed to evaluate CT-based morphometric aortic remodeling parameters, their associated determinants, and mid-term clinical outcomes in a consecutive cohort of patients undergoing TEVAR for acute Stanford type B aortic dissection.

## 2. Materials and Methods

### 2.1. Study Design and Ethical Approval

This retrospective, single-center, observational cohort study was conducted at a tertiary referral center specializing in cardiovascular and endovascular surgery. Clinical, radiological, and procedural data of all consecutive patients who underwent TEVAR for aortic dissection between January 2020 and January 2025 were reviewed. The study was approved by the Institutional Ethics Committee and conducted in accordance with the Declaration of Helsinki and Good Clinical Practice guidelines. Patient consent was waived due to the retrospective design; all data were anonymized prior to analysis. The reporting process followed the STROBE guidelines for observational studies [10].

### 2.2. Patient Selection

**Figure 1:**
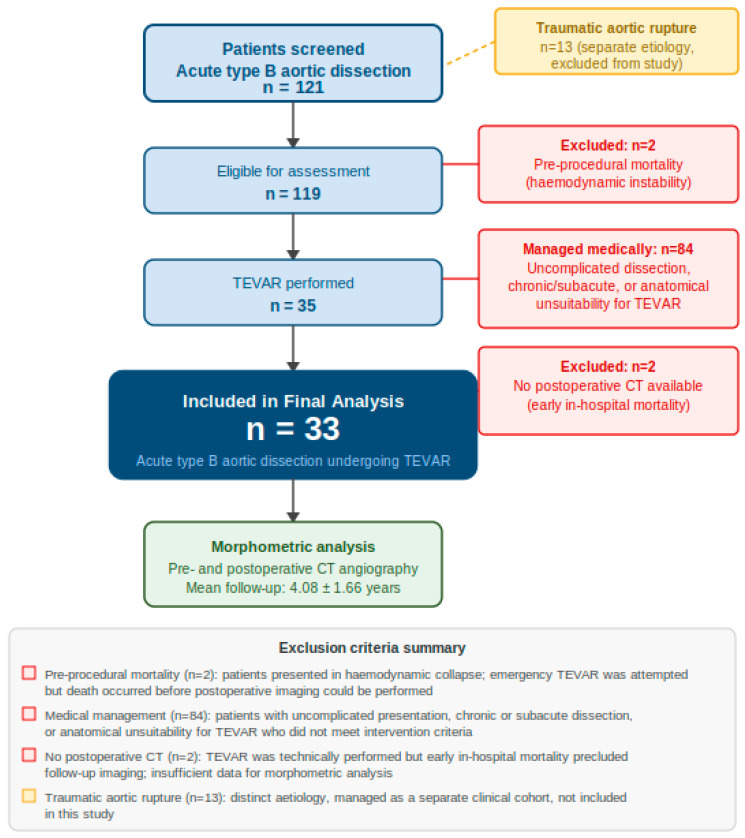
Patient selection flow diagram. TEVAR: thoracic endovascular aortic repair; CT: computed tomography.

Patients were identified through ICD-10 diagnosis codes via the hospital information system. Inclusion criteria were: (i) Stanford type B aortic dissection confirmed by CT angiography; (ii) availability of preoperative and at least one postoperative CT angiography suitable for standardized morphometric measurements; and (iii) acute presentation (symptom onset to intervention ≤ 14 days). Exclusion criteria were: traumatic aortic rupture, inadequate clinical records, or images unsuitable for multiplanar reconstruction (MPR) measurements. All consecutive eligible patients within the study period were included. The flow diagram is presented in Figure 1. Sample size analysis, based on a matched-comparison model for TL and FL diameter changes, indicated a minimum of 28 patients for 80% statistical power at α = 0.05; the cohort of 33 patients satisfied this requirement.

### 2.3. Dissection Classification and Outcome Definitions

Dissections were classified according to the Stanford system. All patients presented acutely (symptom to intervention ≤ 14 days), consistent with current ESC guidelines. Early mortality was defined as in-hospital death or death within 30 days; late mortality as death beyond one year. Endoleak was classified per standard criteria (Types I–IV). Technical success was defined as successful stent–graft deployment with coverage of the primary entry tear and absence of Type I or III endoleak on completion angiography. Secondary intervention was defined as any additional endovascular procedure during follow-up. Complete FL thrombosis was defined as the absence of contrast opacification in the FL on follow-up CT.

### 2.4. Endovascular Technique and Landing Zone Classification

All procedures were performed by a dedicated three-surgeon cardiovascular team, each with experience of more than 50 prior TEVAR cases, in an interventional suite equipped with a fixed GE Innova IGS fluoroscopy system (GE Healthcare, Chicago, IL, USA). Stent–graft selection, sizing, and deployment were based on preoperative CT morphology. All stent–graft systems were the Lifetech Ankura TAA Stent–Graft System (Lifetech Scientific, Shenzhen, China), a self-expanding nitinol device featuring a proximal bare-spring configuration, tip-capture deployment mechanism, and proximal mini-wave design for wall apposition. Stent–graft diameters ranged from 28 to 44 mm, selected with 0–10% oversizing relative to the reference aortic diameter. The proximal landing zone was classified according to the Ishimaru system. Left subclavian artery (LSA) revascularization via carotid-subclavian bypass was performed selectively in Zone 2 patients and in those with a dominant left vertebral artery or prior CABG using the left internal mammary artery (LIMA). Carotid-subclavian bypass was performed exclusively in all Zone 2 landing patients (*n* = 11) as a dedicated first-stage procedure in a separate session prior to the index TEVAR, following the hybrid technique protocol established at our center [11,12]. Preoperative CTA routinely assessed vertebral artery dominance and circle of Willis patency prior to Zone 2 deployment and LSA coverage decisions. Intravascular ultrasound (IVUS) was not used in any case owing to the absence of institutional reimbursement coverage during the study period.

### 2.5. Imaging Protocol and Morphometric Analysis

Contrast-enhanced CT angiography was performed at the institution’s standard protocol. TL, FL, and DA diameters were measured in MPR slices reconstructed perpendicular to the aortic centerline at predefined standard anatomical levels along the descending thoracic aorta. FL diameter was calculated as the difference between the total DA diameter and the TL diameter on the same axial plane. All measurements were performed by experienced cardiovascular surgeons using the institution’s validated radiology workstation software.

### 2.6. Statistical Analysis

Continuous variables were assessed for normality using the Shapiro–Wilk test. Normally distributed variables are presented as mean ± SD; non-normally distributed variables as median with range. Categorical variables are expressed as counts and percentages. Pre- and postoperative diameter changes were analyzed using the paired *t*-test (normal distribution) or Wilcoxon signed-rank test (non-normal distribution), applied to the delta distributions. Between-group differences in continuous variables were assessed using the Mann–Whitney U test; differences in categorical variables were assessed using Fisher’s exact test. Spearman’s rank correlation was used to assess factors associated with aortic remodeling. A two-sided *p* < 0.05 was considered statistically significant. Statistical analyses were performed using SPSS version 27.0 (IBM Corp., Armonk, NY, USA).

## 3. Results

### 3.1. Demographic and Clinical Characteristics

A total of 33 patients were included. Baseline characteristics are summarized in Table 1. Mean age was 53.61 ± 12.08 years (median 55; range 30–85); 27 patients (81.8%) were male. Mean follow-up was 4.08 ± 1.66 years. Hypertension was the predominant comorbidity (97.0%); the most common antihypertensive regimen was a beta blocker combined with a calcium channel blocker (33.3%). Seven patients (21.2%) had prior open cardiac surgery. One patient (3.0%) had genetically confirmed Marfan syndrome. Antiplatelet and anticoagulant therapy were used in 12 (36.4%) and 6 patients (18.2%), respectively.

The most common presenting symptom was back pain alone (33.3%), followed by chest pain (27.3%), combined chest and back pain (15.2%), malperfusion with back pain (18.2%), and abdominal pain (6.1%). Six patients (18.2%) presented with malperfusion syndrome: extremity ischemia in three, mesenteric ischemia in two, and spinal ischemia (paraplegia) in one. Spinal drainage was placed in the patient with preoperative paraplegia. All patients were treated within the acute phase (mean time to intervention 2.73 ± 3.86 days, median 1 day).

### 3.2. Dissection Morphology

Imaging characteristics are presented in Table 2. All 33 patients had acute Stanford type B dissection. Mean dissection length was 44.09 ± 9.71 cm (median 46; range 13–61). Mean preoperative DA diameter was 42.94 ± 6.90 mm (median 41) and mean TL diameter was 9.55 ± 5.91 mm (median 8; range 2–25). Preoperative FL was non-thrombosed in 21 patients (63.6%), partially thrombosed in 9 (27.3%), and completely thrombosed in 3 (9.1%). Aortic wall calcification was present in 12 patients (36.4%). Distal re-entry foci were most commonly infrarenal (75.8%), and a single distal re-entry was identified in 27 patients (81.8%).

### 3.3. Procedural Characteristics

Procedural details are summarized in Table 3. General anesthesia was used in 26 patients (78.8%). Isolated TEVAR was performed in 26 patients (78.8%); the remaining 7 (21.2%) underwent TEVAR combined with adjunctive abdominal aortic bare-metal stenting according to the PETTICOAT (Provisional Extension To Induce Complete Attachment) principle, using Optimed Sinus-XL or Jotec E-XL bare-metal stents deployed at the supracoeliac level. The decision for adjunctive stenting was based on the intraoperative finding of persistent true-lumen collapse or refractory malperfusion syndrome. All stent–graft systems were Lifetech Scientific (Shenzhen, China) [4]. Mean stent–graft length was 22.39 ± 7.28 cm (range 12–38). Zone 2 proximal deployment was performed in 11 patients (33.3%) and Zone 3 in 22 (66.7%). Carotid-subclavian bypass was required in all Zone 2 patients (33.3%), reflecting the need for adequate proximal seal zone and LIMA protection in patients with prior CABG. Mean ICU stay was 1.88 ± 3.22 days and mean total hospital stay was 8.61 ± 5.25 days. Intravascular ultrasound (IVUS) was not used in any case owing to the absence of institutional reimbursement coverage for this technology during the study period.

### 3.4. Clinical Outcomes

Early and follow-up outcomes are presented in Table 4. Mean clinical follow-up was 4.08 ± 1.66 years, defined as the interval from the index procedure to the last outpatient contact. Radiological follow-up by CT angiography was conducted independently: all 33 patients (100%) underwent first postoperative CT angiography (CT1) at a median of 25 days following the index procedure (institutional protocol: first month). A second CT angiography (CT2) was performed in 27 patients (81.8%) at a median of 5.4 months (institutional protocol: sixth month); the remaining six patients had either died before the scheduled examination or did not complete imaging follow-up.

Postoperative complications occurred in a total of nine patients. Renal complications were observed in four patients (12.1%): elevated creatinine in two (6.1%) and dialysis-requiring acute kidney injury in two (6.1%). Wound infection occurred in three patients (9.1%), neurological complications in one patient (3.0%), and gastrointestinal complications in two (6.1%). Neurological events occurred in two patients: one developed a new post-procedural ischaemic stroke (left frontal infarction confirmed on MRI), recorded as a de novo procedural complication (*n* = 1, 3.0%); the second patient experienced spinal cord ischaemia with bilateral lower-limb plegia attributable to the pre-existing malperfusion syndrome, captured in the malperfusion subgroup rather than the procedural neurological complication category.

Endoleak developed in nine patients (27.3%). Type II was the most common subtype (*n* = 5, 15.2%): four cases were attributable to retrograde left subclavian artery filling in Zone 2 patients, managed with Amplatzer vascular plug occlusion performed exclusively upon confirmed detection of LSA-related Type II endoleak on follow-up CT angiography—no prophylactic embolisation was performed at the index procedure; one lumbar artery source was managed conservatively. Type Ia endoleak (*n* = 3, 9.1%) was treated with proximal TEVAR extension. Type IV endoleak (*n* = 1, 3.0%) resolved spontaneously. Zone 2 landing was significantly associated with higher endoleak rates (54.5% vs. 13.6% in Zone 3; *p* = 0.033).

Complete FL thrombosis developed in 19 patients (57.6%) during follow-up, while the FL remained patent in 12 (36.4%) and partially thrombosed in 2 (6.1%). Secondary intervention was required in 13 patients (39.4%). TEVAR extension was performed in eight patients (24.2%) for the following indications: Type Ia endoleak in three patients, proximal pseudoaneurysm formation in one patient, and progressive aortic enlargement at the distal stent–graft margin in four patients. LSA Amplatzer vascular plug occlusion was performed in four patients (12.1%) upon confirmed LSA-related Type II endoleak, and combined Amplatzer occlusion with TEVAR extension in one patient (3.0%).

No cases of stent–graft-induced new entry (SINE), either proximal or distal, were identified during the follow-up period.

**Figure 2:**
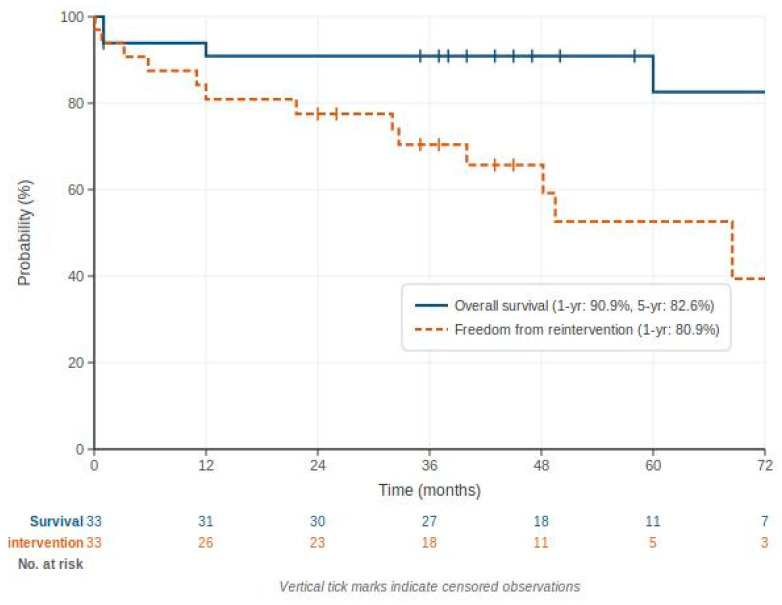
Kaplan–Meier estimates of overall survival (solid line, 1 year: 90.9%, 5 years: 82.6%) and freedom from secondary intervention (dashed line, 1 year: 80.9%) in 33 patients undergoing TEVAR for acute Stanford type B aortic dissection. Tick marks indicate censored observations. Numbers at risk are shown below the time axis.

Total mortality was 12.1% (*n* = 4). Early aortic-related mortality occurred in two patients (6.1%), both presenting with malperfusion (mesenteric ischemia and rupture). Late non-aortic mortality occurred in two patients (6.1%): one from COPD exacerbation with pneumonia at one year, and one from septic acute renal failure at five years (Figure 2). No statistically significant factor associated with mortality was identified on univariable analysis, reflecting limited statistical power given the small number of events. 

Postoperative re-entry foci were identified in 25 patients (75.8%): most commonly infrarenal (*n* = 22, 66.7%), followed by thoracic aortic (*n* = 2, 6.1%) and suprarenal (*n* = 1, 3.0%); no re-entry was detected in 8 patients (24.2%).

### 3.5. Morphometric Remodeling Analysis

Shapiro–Wilk testing confirmed normal distribution for TL delta (W = 0.954, *p* = 0.176) and FL delta (W = 0.957, *p* = 0.213), supporting the paired *t*-test for these variables. DA delta was non-normally distributed (W = 0.902, *p* = 0.006), and the Wilcoxon signed-rank test was applied. Results are presented in Table 5.

DA diameter remained stable (42.94 ± 6.90 mm preoperatively vs. 42.03 ± 9.46 mm postoperatively; Δ = −0.91 ± 8.21 mm, median −1 [−13 to +28]; *p* = 0.323). TL diameter increased from 9.55 ± 5.91 mm to 28.30 ± 5.49 mm (+18.76 ± 8.83 mm, median +20; *p* < 0.001), and FL diameter decreased from 33.39 ± 6.76 mm to 11.48 ± 8.97 mm (−21.91 ± 9.53 mm, median −23; *p* < 0.001). The stability of overall aortic diameter in the context of significant intraluminal redistribution reflects a favorable structural response without aneurysmal degeneration.

### 3.6. Factors Associated with Endoleak, Reintervention, FL Thrombosis, and Remodeling

Among continuous variables, a larger preoperative DA diameter was significantly associated with endoleak development (median 47.0 vs. 40.0 mm in endoleak-positive vs. endoleak-negative patients; *p* = 0.049). Among categorical variables, Zone 2 landing was the only statistically significant factor associated with endoleak (54.5% vs. 13.6% in Zone 3; *p* = 0.033). No significant factor associated with secondary intervention or FL complete thrombosis was identified on univariable analysis, likely reflecting limited statistical power given the small number of events. Univariable factors associated with endoleak are presented in Table 6.

Spearman’s correlation analysis identified preoperative TL diameter as the factor most strongly associated with postoperative TL expansion (r = −0.724, *p* < 0.001): a narrower baseline TL was associated with greater absolute remodeling gain, consistent with the greater expansion potential of a severely compressed lumen. Stent–graft length was positively correlated with TL delta (r = +0.522, *p* = 0.002), suggesting that longer aortic coverage promotes greater hemodynamic redistribution. Preoperative FL size was negatively correlated with FL regression (r = −0.489, *p* = 0.004), indicating that a larger initial FL undergoes greater absolute shrinkage following TEVAR. Spearman’s correlation results are summarized in Table 7.

## 4. Discussion

We evaluated morphometric aortic remodeling and mid-term clinical outcomes in 33 consecutive patients undergoing TEVAR for acute Stanford type B aortic dissection. True lumen (TL) diameter increased by 18.76 ± 8.83 mm (*p* < 0.001) and false lumen (FL) diameter decreased by 21.91 ± 9.53 mm (*p* < 0.001), while total descending aortic diameter remained stable (Δ = −0.91 ± 8.21 mm; *p* = 0.323)—a pattern of intraluminal redistribution without net aortic wall dilatation. Several procedural and morphological variables determined the magnitude of the remodeling response and the downstream clinical course.

### 4.1. Rationale for Acute-Phase Intervention and Indications

One aspect of this cohort that differs from many published series is the exclusive use of acute-phase (≤14 days) TEVAR. Some groups favor deferral to the subacute window (15–90 days) to allow tissue maturation and reduce procedural risk [13,14].

All 33 patients in this cohort met one or more evidence-based indications for urgent or emergency endovascular intervention, precluding deferral to the subacute window. These indications included: malperfusion syndrome (visceral, extremity, or spinal; *n* = 6, 18.2%), threatening aortic rupture (*n* = 2, 6.1%), refractory hypertension unresponsive to at least three antihypertensive agents including beta blockers (*n* = 15, 45.5%), persistent or recurrent pain despite adequate medical therapy (*n* = 11, 33.3%), and rapidly expanding aortic diameter (≥40 mm with evidence of early false lumen pressurization) on admission CT angiography (*n* = 8, 24.2%). These fall into the ‘complicated’ and ‘high-risk uncomplicated’ categories as defined by the SVS/STS 2020 guidelines and the 2024 ESC Aorta Guidelines, for which early endovascular intervention is supported by class I–IIa evidence [2,3].

The debate surrounding acute versus subacute TEVAR timing is well established in the literature. Several meta-analyses and comparative series have demonstrated that acute-phase TEVAR (≤14 days) carries higher short-term complication rates—including elevated stroke incidence (HR 2.63 vs. subacute) and 30-day mortality—compared with subacute intervention in uncomplicated dissections [13]. However, this comparison conflates two very different patient groups: stable, uncomplicated dissection, where deferral is genuinely possible, and complicated or hemodynamically unstable presentations, where waiting is not. In our cohort, the decision to intervene acutely was not elective—it was dictated by the clinical urgency of each presentation. Subacute-phase timing applies specifically to stable, uncomplicated high-risk patients where deferral does not add clinical risk. In the presence of end-organ ischemia, an evolving hemothorax, or refractory pain signaling impending rupture, the operative risk of delay far outweighs that of acute intervention.

Refractory pain deserves particular attention as an indication for urgent TEVAR, as it is sometimes underappreciated relative to malperfusion. Persistent pain despite adequate analgesia and blood pressure control reflects ongoing intimal shear stress, false lumen pressurization, and aortic wall tension—all of which herald impending progression to rupture or extension [3,14]. In our series, 11 patients (33.3%) underwent acute TEVAR primarily for this indication. Current guidelines from five major societies (ESC, SVS/STS, ESVS, JVS, AHA/ACC) consistently classify refractory pain as a high-risk feature that mandates early intervention, with several categorizing it on par with acute malperfusion in terms of urgency [2,3,14].

### 4.2. Aortic Remodeling: Magnitude, Mechanism, and Associated Factors

The magnitude of TL expansion observed in this cohort (+18.76 ± 8.83 mm, median +20 mm) exceeds values reported in uncomplicated dissection trials. The ADSORB trial reported a median TL gain of approximately +8 mm at one year in uncomplicated type B dissection [15]. The VIRTUE Registry documented greater remodeling in acute compared with subacute or chronic presentations [8]. The larger TL expansion in our cohort, despite a complicated clinical profile, likely reflects the severely compressed baseline TL dimensions (mean 9.55 mm, median 8 mm)—in line with the expansion potential quantified by Spearman’s correlation (r = −0.724, *p* < 0.001): a severely compressed TL carries greater elastic recoil reserve.

The strong negative Spearman’s correlation between preoperative TL diameter and postoperative TL expansion (r = −0.724, *p* < 0.001) is the most clinically relevant observation of this analysis: patients with the most severely compressed TL at baseline showed the greatest absolute TL expansion after TEVAR. Physiologically, this reflects the elasticity gradient: a highly compressed TL has maximal potential energy for expansion once the compressive FL pressure is relieved by primary tear occlusion [16]. Conversely, a less compressed TL has less room to expand proportionally. This relationship has been implied in prior series [5,6], but, to our knowledge, has not been formally quantified with this magnitude of correlation in a standardized MPR-based measurement study. From a clinical standpoint, a severely compressed true lumen may help identify patients who could derive greater remodeling benefit from early TEVAR; however, this observation should be considered hypothesis-generating and requires confirmation in larger prospective studies before it can be used to guide treatment timing decisions.

The positive correlation between stent–graft length and TL expansion (r = +0.522, *p* = 0.002) provides further mechanistic insight. Longer aortic coverage occludes a greater number of primary and secondary entry tears, resulting in more complete FL depressurization along the stented segment and consequently greater TL gain. This supports the principle of maximizing anatomical coverage when technically feasible—a strategy sometimes described as ‘total thoracic coverage’—provided that the risk of spinal cord ischemia is mitigated through appropriate protective measures, including cerebrospinal fluid drainage, staged procedures, and preservation of collateral spinal circulation [2]. In our cohort, the selective application of spinal drainage in the patient with preoperative paraplegia and the exclusive use of Lifetech Scientific stent–graft systems of sufficient length likely contributed to this observed association.

The stability of the total descending aortic diameter (Δ = −0.91 mm; *p* = 0.323) should not be interpreted as an absence of remodeling but rather as a favorable structural response: intraluminal hemodynamic redistribution occurring without net aortic wall dilatation. Progressive aortic enlargement—the hallmark of aneurysmal degeneration—was absent in this cohort over a mean follow-up of 4.08 years, suggesting that effective TL restoration suppressed the chronic FL-mediated mural stress that drives late aneurysmal degeneration [17].

### 4.3. False Lumen Thrombosis and the Significance of Baseline FL Morphology

Complete FL thrombosis was achieved in 57.6% of patients, with the FL remaining patent in 36.4% and partially thrombosed in 6.1%. The rate of complete FL thrombosis in this cohort is lower than the 91.3% reported at five years in the INSTEAD-XL TEVAR arm [7] and the 73% reported at three years in the VIRTUE acute subgroup [8]. Several factors may account for this discrepancy. First, the high proportion of patients with non-thrombosed FL at baseline (63.6%) indicates that this morphological subtype has the greatest dynamic flow and the largest number of re-entry foci, making spontaneous thrombosis unlikely even after adequate proximal stent–graft coverage [18,19]. Second, the concentration of distal re-entry foci at the infrarenal level (75.8%)—beyond the stented segment—maintains persistent FL filling from below regardless of proximal entry tear coverage. Third, the inclusion of complex and high-risk patients with extensive dissection (mean length: 44.09 cm) indicates that complete FL exclusion with a single thoracic stent–graft is anatomically insufficient in most cases.

The prognostic significance of preoperative FL thrombosis pattern merits separate discussion. Partial thrombosis of the FL—present in 27.3% of our cohort preoperatively—carries a paradoxically worse long-term prognosis compared with either complete thrombosis or a fully patent FL—a finding reported in the IRAD dataset [19,20]. This counterintuitive relationship reflects the fact that partial thrombosis creates a localized pressure gradient that may paradoxically increase FL wall tension at the transition zone. While the small number of patients with preoperative complete thrombosis (*n* = 3, 9.1%) precludes formal subgroup analysis, the descriptive data suggest that this group had the least TL expansion (median Δ = +14 mm) compared with non-thrombosed (median Δ = +22 mm) and partial thrombosis groups (median Δ = +20 mm), in keeping with a reduced hemodynamic benefit from TEVAR when the FL is already thrombosed—an area warranting investigation in larger cohorts.

### 4.4. Landing Zone Selection and Endoleak Prevention

The proximal landing Zone 2 was significantly associated with higher endoleak rates than Zone 3 (54.5% vs. 13.6%; *p* = 0.033). The predominant endoleak mechanism in Zone 2 patients was retrograde filling of the FL via the left subclavian artery (*n* = 4 of 5 Type II endoleaks), reflecting the well-documented risk of LSA-sourced endoleak when Zone 2 deployment covers the subclavian ostium [21,22]. This complication was successfully managed in all four cases with Amplatzer vascular plug occlusion of the left subclavian artery, facilitated by the pre-existing carotid-subclavian bypass performed at the time of the index procedure.

Our institutional strategy for Zone 2 TEVAR—routine carotid-subclavian bypass performed as the first stage of a hybrid procedure—served a dual purpose: it ensured cerebral and upper-extremity perfusion safety during subclavian coverage and provided a conduit for planned LSA occlusion when a Type II endoleak developed during follow-up. This planned hybrid approach avoids the neurological and ischemic risks of emergency LSA occlusion without revascularization and follows current guideline recommendations for routine preoperative subclavian revascularization in patients requiring Zone 2 coverage [2]. A potential refinement for future practice is routine simultaneous LSA embolization at the time of the index TEVAR procedure in confirmed Zone 2 cases, rather than deferring occlusion to a secondary intervention. This strategy would eliminate the LSA as a retrograde endoleak source from the outset, potentially reducing the overall reintervention rate in this subgroup. This approach is contingent, however, on confirmed carotid-subclavian bypass patency and adequate posterior circulation reserve, as deliberate LSA occlusion without a functioning revascularization carries a documented risk of posterior stroke and spinal cord ischemia [22]. When these safety conditions are met, upfront LSA occlusion at the index procedure may represent the preferred strategy in confirmed Zone 2 TEVAR. The high carotid-subclavian bypass rate (33.3%) in our series reflects the specific anatomical characteristics of our cohort: the extension of the dissection into the LSA in the majority of Zone 2 cases, combined with the presence of prior CABG using LIMA in several patients for whom subclavian coverage without revascularization would have precipitated graft ischemia.

It is also noteworthy that dedicated branched aortic arch endografts with integrated left subclavian artery branches—such as the Castor single-branched endoprosthesis (MicroPort, Shanghai, China) or the Gore Thoracic Branch Endoprosthesis (W.L. Gore, Flagstaff, AZ, USA)—were not used in this series. These two platforms differ in key technical and anatomical respects: the Castor device incorporates a single side branch for the LSA and is designed for sequential deployment in Zone 2, while the Gore TBE uses a single-lumen branched configuration with broader diameter compatibility but more demanding proximal sealing zone requirements. Both devices avoid the need for surgical bypass revascularisation and may be combined with dissection-specific adjunctive strategies such as PETTICOAT or STABILIZE to address persistent distal false-lumen perfusion in complicated type B dissection [23]. Their use was precluded in our center by the absence of national reimbursement coverage and limited institutional experience with these platforms during the study period. As these devices become more widely available, they may represent a preferred strategy for Zone 2 TEVAR in centers with appropriate anatomical selection and technical expertise.

The significant association between preoperative DA diameter and endoleak development (median 47.0 vs. 40.0 mm in endoleak-positive vs. endoleak-negative patients; *p* = 0.049) likely reflects the greater challenge of achieving adequate stent–graft apposition in larger, more distorted dissection anatomy. Larger aortic diameters are associated with greater variability in luminal geometry, calcification, and intimal flap rigidity—all of which compromise the circumferential seal of the stent–graft and predispose to both Type Ia and Type II endoleak. This finding supports the argument for more careful preprocedural planning and, in patients with DA diameters exceeding 45 mm at presentation, potentially more aggressive oversizing strategies (within safe limits).

### 4.5. Procedural Measures to Reduce Retrograde Type A Aortic Dissection Risk

Retrograde type A aortic dissection (RTAD) is the most feared procedural complication of TEVAR for type B dissection, with a reported incidence of 2–5% across contemporary series [24]. No RTAD occurred in this cohort, a result we attribute to several deliberate technical precautions applied consistently throughout the study period.

The absence of SINE in this series is consistent with the deliberate policy of minimal stent–graft oversizing (0–10%). Distal SINE after TEVAR for acute-phase dissection has been reported in approximately 4% of cases in the literature, with oversizing identified as the primary modifiable risk factor; higher rates observed in chronic dissection series (~13%) further support conservative sizing in the acute setting.

First, minimal stent–graft oversizing (0–10% of the reference aortic diameter) was maintained in all acute cases. This strategy differs from the more aggressive oversizing applied in aneurysmal disease (typically 15–20%) and is supported by multiple studies demonstrating that oversizing exceeding 20% significantly increases radial forces on the fragile, edematous intima of the acute dissection aortic wall, creating the mechanical conditions for retrograde propagation [24]. The physiological rationale is straightforward: in acute dissection, the aortic wall is already mechanically compromised; oversizing adds circumferential stress to a fragile intima at the proximal stent–graft edge precisely where the mobile, non-apposed intimal flap transitions to the ascending aorta.

Second, balloon dilatation of the deployed stent–graft was avoided unless compelling evidence of persistent endoleak or insufficient TL expansion was present on completion angiography. Compliant balloon molding imposes focal radial forces concentrated at the proximal edge of the stent–graft—a particularly vulnerable zone in acute dissection where the flap is least adherent. This conservative approach is consistent with ACC/AHA recommendations [2].

Third, careful wire management was employed throughout each procedure. After transfemoral access, wire positioning within the true lumen was confirmed by intravascular guidance prior to stent–graft delivery. Excessive manipulation of stiff delivery systems and guidewires within the partially obstructed TL was minimized. Sudden catheter repositioning against a mobile intimal flap can generate focal intimal disruption and retrograde propagation, particularly in the acute phase when the flap has not yet formed adhesions to the outer aortic wall.

Fourth, the deployment speed of the stent–graft was controlled to minimize the hydraulic ‘water hammer’ effect—the transient pressure wave generated by rapid stent–graft expansion—which has been proposed as a mechanism of proximal intimal injury during deployment, particularly when the proximal landing zone is in close proximity to the aortic arch.

Fifth, the landing zone was carefully selected to avoid landing in severely angulated, calcified, or dilated segments. The gothic arch configuration—characterized by a pointed apex with steep angulation between the ascending and descending aorta—creates a mismatch between the rigid stent–graft and the curved aortic wall at the proximal landing zone, generating eccentric radial forces that predispose to RTAD [24]. In our cohort, 22 patients (66.7%) were deployed in Zone 3, which provides a more favorable, relatively straight, and disease-free proximal landing compared with Zone 2.

### 4.6. Malperfusion Subgroup: Paradoxical Remodeling Advantage

In the malperfusion subgroup (*n* = 6), paradoxically favorable morphometric remodeling was observed compared with non-malperfusion patients: median TL expansion was +25.5 mm versus +19.0 mm, and FL regression was −29.5 mm versus −22.0 mm. No endoleak occurred in any malperfusion patient (0% vs. 33.3% in the non-malperfusion group). These findings may be explained by several mechanisms. Malperfusion patients typically present with the most severely compressed TL—indeed, the expansion potential of a severely compressed lumen is most prominently realized in this group. The acuity and severity of TL compromise in malperfusion cases also mandate more technically precise stent–graft deployment aimed at complete entry tear coverage and maximal TL restoration, leaving less margin for technical suboptimality. All malperfusion patients in our cohort were treated with Zone 3 landing—which inherently carries a lower endoleak risk—partly because malperfusion cases tend to have the primary entry tear in the proximal descending aorta, which is amenable to Zone 3 coverage.

No secondary interventions were required in the malperfusion group (0% vs. 48.1% in the non-malperfusion group), further suggesting that once acute organ perfusion is restored by TEVAR in these patients, the morphological trajectory is uniformly favorable. Similar findings have been reported in a dedicated series of complicated acute type B dissections, which also included a malperfusion subset [25]. Importantly, our two early mortalities occurred in the malperfusion subgroup (mesenteric ischemia and rupture)—reflecting the mortality risk intrinsic to this presentation, independent of procedural technical success. The irreversible visceral ischemia preceding TEVAR in these cases, rather than procedural failure, drove the fatal outcome.

### 4.7. Secondary Interventions: A Marker of Disease Dynamism, Not Technical Failure

Secondary intervention was required in 13 patients (39.4%), with TEVAR extension being the most common procedure (*n* = 8, 24.2%). While this rate may appear elevated relative to uncomplicated dissection series, it must be contextualized within three realities. First, the cohort consists entirely of complex or high-risk presentations with extensive dissection (mean length 44.09 cm), multiple re-entry foci (75.8% with identifiable postoperative re-entry), and high rates of Zone 2 deployment—all established risk factors for reintervention. Second, the single stent–graft approach—which addressed the proximal entry tear but did not exclude the abundant infrarenal re-entry foci (66.7%)—is mechanistically insufficient to achieve complete FL exclusion in most patients. The persistence of infrarenal re-entry maintains FL pressurization via retrograde flow, driving progressive dissection and necessitating staged endovascular management. Third, the 4.08-year mean follow-up represents a medium-term surveillance window that naturally captures late-appearing complications as the dissection continues to evolve biologically [26].

These observations support a conceptual framework in which TEVAR for complicated type B dissection should be viewed as the first stage of a longitudinal staged repair strategy rather than a definitive, single-session cure. The goal of the index procedure is to resolve immediate life-threatening complications and initiate the remodeling process; subsequent procedures are planned responses to the ongoing aortic disease process. This staged approach—analogous to the PETTICOAT (Provisional Extension To Induce Complete Attachment) concept for addressing distal re-entry—is increasingly endorsed by international guidelines and represents current best practice for complex dissections extending into the abdominal aorta [2,3].

### 4.8. Comparison with Contemporary Series and Landmark Trials

Comparing these results with major randomized trials requires attention to differences in patient selection [27]. The INSTEAD-XL trial enrolled exclusively uncomplicated type B dissections—a population that differs fundamentally from ours in risk profile and clinical urgency [7]. The ADSORB trial similarly enrolled uncomplicated cases and performed TEVAR in the subacute phase [15]. The VIRTUE Registry is perhaps the most comparable series, as it included acute, subacute, and chronic presentations; its acute subgroup showed the most pronounced remodeling, in line with our findings [8]. Our cohort’s complete FL thrombosis rate (57.6%) lies between the VIRTUE acute subgroup (~70%) and chronic subgroup (~40%), despite a predominantly complicated presentation, suggesting that the beneficial remodeling effect of TEVAR extends to complicated dissections when performed with appropriate technical precision.

Our mortality rate (12.1%) is consistent with reported outcomes for complicated type B dissection requiring urgent TEVAR. Contemporary large series report 30-day mortality of 5–15% in complicated dissection, with higher rates in cases involving rupture or mesenteric ischemia [25]. Both early deaths in our series occurred in patients with the highest-risk presentations (rupture and mesenteric ischemia), and neither was attributable to procedural technical failure. Late mortality (6.1%), both from non-aortic causes, reflects the general cardiovascular comorbidity burden of this population rather than dissection-specific disease progression—a favorable finding suggesting effective long-term aortic stabilization.

### 4.9. Statistical Power and Interpretation of Null Findings

The lack of statistically significant univariable factors associated with secondary intervention or FL complete thrombosis does not rule out biologically meaningful associations—it reflects the capacity of *n* = 33 to detect only large effect sizes (statistical power < 80% for odds ratios < 3 with event frequencies of 30–60%). A priori power calculations for these secondary endpoints were not performed, as the study was powered for the primary morphometric comparison. The borderline *p*-values observed for several variables (prior cardiac surgery *p* = 0.068, malperfusion *p* = 0.060, and younger age *p* = 0.055 for reintervention) are hypothesis-generating and warrant prospective evaluation in larger cohorts. We deliberately refrain from multivariable modeling given the event-to-variable ratio constraints, a methodological restraint recommended by established statistical guidance for binary outcomes [10].

Several limitations warrant acknowledgement. Systematic screening for SINE relied on routine CT angiography surveillance; subclinical distal SINE without hemodynamic consequence may theoretically have been missed in the absence of dedicated SINE-specific imaging protocols. First, the retrospective single-center design and consecutive series of 33 patients limit generalizability; referral patterns and institutional expertise of a tertiary center may not reflect broader practice. Second, the sample size, while adequate for the primary morphometric comparison, was underpowered for multivariable modeling of secondary outcomes; all analyses were univariable and should be considered hypothesis-generating. Third, aortic remodeling was quantified using single-plane CT diameter measurements rather than volumetric FL analysis, which would capture spatial remodeling more precisely. Fourth, the exclusive use of a single stent–graft platform (Lifetech Scientific) precludes device-comparative conclusions; observer variability for morphometric measurements was not formally assessed; and the absence of a contemporaneous control group prevents direct comparison of acute versus subacute TEVAR timing in this complicated cohort.

## 5. Conclusions

In this cohort, TEVAR resulted in marked TL expansion and FL regression without net aortic enlargement in all 33 patients with acute Stanford type B aortic dissection. Narrower baseline TL diameter and longer stent–graft coverage were each associated with greater remodeling gain. Proximal zone 2 deployment was associated with a significantly higher endoleak burden, underscoring the importance of careful preprocedural planning and systematic postprocedural imaging follow-up.

## Figures and Tables

**Table 1:** Baseline demographic and clinical characteristics (*n* = 33).

Variable	Value
Age (years), mean ± SD (median)	53.61 ± 12.08 (55) [30–85]
Male sex, n (%)	27 (81.8)
Follow-up (years), mean ± SD	4.08 ± 1.66
Body weight (kg), mean ± SD	80.79 ± 11.87
BMI (kg/m^2^), mean ± SD	28.94 ± 5.37
**Comorbidities**	
Hypertension	32 (97.0)
Diabetes mellitus	3 (9.1)
Chronic kidney disease	3 (9.1)
COPD	2 (6.1)
Coronary artery disease	4 (12.1)
Cigarette use	7 (21.2)
Connective tissue disease (Marfan, confirmed)	1 (3.0)
Prior open cardiac surgery	7 (21.2)
**Medication use**	
Antiplatelet therapy	12 (36.4)
ASA	11 (33.3)
Clopidogrel	1 (3.0)
Anticoagulant therapy	6 (18.2)
Warfarin	5 (15.2)
DOAC	1 (3.0)
Concurrent antiplatelet + anticoagulant	4 (12.1)
Statin	4 (12.1)
**Presenting symptoms**	
Back pain	11 (33.3)
Chest pain	9 (27.3)
Chest and back pain	5 (15.2)
Malperfusion + back pain	6 (18.2)
Abdominal pain	2 (6.1)
**Malperfusion syndrome—6 (18.2%)**	
Extremity ischemia	3 (9.1)
Mesenteric ischemia	2 (6.1)
Spinal ischemia (paraplegia)	1 (3.0)
Time to intervention (days), mean ± SD (median)	2.73 ± 3.86 (1) [0–14]

ASA: acetylsalicylic acid; DOAC: direct oral anticoagulant; COPD: chronic obstructive pulmonary disease; BMI: body mass index.

**Table 2:** Dissection morphology and imaging characteristics (*n* = 33).

Variable	Value
Stanford type B, n (%)	33 (100)
Dissection length (cm), mean ± SD (median)	44.09 ± 9.71 (46) [13–61]
Preop DA diameter (mm), mean ± SD (median)	42.94 ± 6.90 (41) [32–55]
Preop TL diameter (mm), mean ± SD (median)	9.55 ± 5.91 (8) [2–25]
**False lumen morphology**	
Non-thrombosed	21 (63.6)
Partially thrombosed	9 (27.3)
Completely thrombosed	3 (9.1)
Aortic wall calcification, n (%)	12 (36.4)
**Distal re-entry location**	
Infrarenal	25 (75.8)
Suprarenal	5 (15.2)
Thoracic aorta	3 (9.1)
**Number of distal re-entries**	
Single	27 (81.8)
Multiple (≥2)	6 (18.2)

DA: descending aorta; TL: true lumen; Preop: preoperative.

**Table 3:** Procedural and perioperative details (*n* = 33).

Variable	Value
**Type of anesthesia**	
General	26 (78.8)
Spinal	4 (12.1)
Local	3 (9.1)
**Stent–graft type**	
Isolated TEVAR	26 (78.8)
TEVAR + abdominal BMS	7 (21.2)
Stent–graft length (cm), mean ± SD (median)	22.39 ± 7.28 (23) [12–38]
**Proximal landing zone (Ishimaru)**	
Zone 2	11 (33.3)
Zone 3	22 (66.7)
Carotid-subclavian bypass, n (%)	11 (33.3)
**Concomitant procedures**	
Iliac stent	1 (3.0)
Right renal artery stent	1 (3.0)
SMA stent	1 (3.0)
SMA + left renal artery stent	1 (3.0)
ICU stay (days), mean ± SD (median)	1.88 ± 3.22 (1) [0–14]
Total hospital stay (days), mean ± SD (median)	8.61 ± 5.25 (6) [1–23]
Perioperative transfusion, n (%)	3 (9.1)

TEVAR: thoracic endovascular aortic repair; BMS: bare-metal stent; SMA: superior mesenteric artery.

**Table 4:** Early and follow-up clinical outcomes (*n* = 33).

Variable	n (%)
Postoperative CT1 performed	33 (100)
Postoperative CT2 performed	27 (81.8)
**Postoperative complications**	
Renal complication (total)	4 (12.1)
Elevated creatinine	2 (6.1)
Need for dialysis	2 (6.1)
Wound infection	3 (9.1)
Neurological complication	1 (3.0)
Gastrointestinal complication	2 (6.1)
**Endoleak—9 (27.3%)**	
Type Ia	3 (9.1)
Type II	5 (15.2)
Type IV	1 (3.0)
**Postoperative re-entry**	
None	8 (24.2)
Infrarenal	22 (66.7)
Suprarenal	1 (3.0)
Thoracic aorta	2 (6.1)
**False lumen status at follow-up**	
Completely thrombosed	19 (57.6)
Non-thrombosed (patent)	12 (36.4)
Partially thrombosed	2 (6.1)
**Secondary interventions—13 (39.4%)**	
TEVAR extension	8 (24.2)
LSA Amplatzer occlusion	4 (12.1)
LSA Amplatzer occlusion + TEVAR extension	1 (3.0)
**Mortality—4 (12.1%)**	
Early mortality (aortic, ≤30 days)	2 (6.1)
Late mortality (non-aortic, >1 year)	2 (6.1)

LSA: left subclavian artery; CT: computed tomography.

**Table 5:** Preoperative and postoperative morphometric comparison (*n* = 33).

Variable	Preoperative Mean ± SD (Median) [Range]	Postoperative Mean ± SD (Median) [Range]	Δ Mean ± SD (95% CI)	*p*-Value
DA (mm)	42.94 ± 6.90 (41) [32–55]	42.03 ± 9.46 (39) [33–73]	−0.91 ± 8.21 (95% CI: −3.8 to 2.0)	0.323 †
TL (mm)	9.55 ± 5.91 (8) [2–25]	28.30 ± 5.49 (28) [18–40]	+18.76 ± 8.83 (95% CI: 15.6 to 21.9)	<0.001 ‡
FL (mm)	33.39 ± 6.76 (33) [14–49]	11.48 ± 8.97 (10) [1–29]	−21.91 ± 9.53 (95% CI: −25.3 to −18.5)	<0.001 ‡

DA: descending aortic diameter; TL: true lumen diameter; FL: false lumen diameter (= DA − TL at same axial level). † Wilcoxon signed-rank test (DA delta non-normal: Shapiro–Wilk W = 0.902, *p* = 0.006). ‡ Paired samples *t*-test (TL delta: SW *p* = 0.176; FL delta: SW *p* = 0.213—normal distribution).

**Table 6:** Univariable analysis of factors associated with endoleak (*n* = 33; endoleak *n* = 9).

Variable	Endoleak+ (*n* = 9)	Endoleak− (*n* = 24)	*p*-Value	Significance
**Continuous variables (Mann–Whitney U)**				
Age (years), median	49.0	55.0	0.531	—
DA preop (mm), median	47.0	40.0	0.049	*
TL preop (mm), median	8.0	8.0	0.855	—
FL preop (mm), median	35.0	32.5	0.321	—
Dissection length (cm), median	45.0	46.0	0.394	—
Stent–graft length (cm), median	23.0	18.0	0.197	—
**Categorical variables (Fisher’s exact)**				
Aortic calcification, n/N	2/12	7/21	0.429	—
Preop partial FL thrombosis, n/N	3/9	6/24	0.677	—
Landing Zone 2, n/N	6/11	3/22	0.033	*
Malperfusion syndrome, n/N	0/6	9/27	0.156	—
Multiple re-entry foci, n/N	2/6	7/27	1.000	—
Prior cardiac surgery, n/N	4/7	5/26	0.068	—

DA: descending aorta; TL: true lumen; FL: false lumen; * *p* < 0.05. Mann–Whitney U and Fisher’s exact results reported as median (IQR); 95% confidence intervals not computed for non-parametric comparisons.

**Table 7:** Spearman’s correlation: factors associated with aortic remodeling.

Variable	TL Δ r	TL Δ *p*	FL Δ r	FL Δ *p*
Age	−0.273	0.124	+0.121	0.503
Preop DA diameter	−0.402	0.020 *	−0.036	0.843
Preop TL diameter	−0.724	<0.001 *	+0.469	0.006 *
Preop FL diameter	+0.188	0.296	−0.489	0.004 *
Dissection length (cm)	+0.313	0.076	−0.316	0.073
Stent–graft length (cm)	+0.522	0.002 *	−0.301	0.089
Follow-up (years)	+0.204	0.256	−0.044	0.809

TL: true lumen; FL: false lumen; Δ: postoperative−preoperative change; * *p* < 0.05.

## Data Availability

The data presented in this study are available on request from the corresponding author.

## Abbreviations

The following abbreviations are used in this manuscript:

TEVAR	Thoracic Endovascular Aortic Repair
TL	True Lumen
FL	False Lumen
DA	Descending Aorta
CTA	Computed Tomography Angiography
MPR	Multiplanar Reconstruction
ICU	Intensive Care Unit
CABG	Coronary Artery Bypass Grafting
LIMA	Left Internal Mammary Artery
LSA	Left Subclavian Artery
SD	Standard Deviation
COPD	Chronic Obstructive Pulmonary Disease
RTAD	Retrograde Type A Aortic Dissection
